# Effect of Bionic Crab Shell Attitude Parameters on Lift and Drag in a Flow Field

**DOI:** 10.3390/biomimetics9020081

**Published:** 2024-01-29

**Authors:** Shihao Hu, Xi Chen, Jiawei Li, Peiye Yu, Mingfei Xin, Biye Pan, Sicen Li, Qinyun Tang, Liquan Wang, Mingxuan Ding, Kaixin Liu, Zhaojin Liu

**Affiliations:** 1 College of Mechanical and Electrical Engineering, Harbin Engineering University, Harbin 150001, China; jhjsb@hrbeu.edu.cn (S.H.); ihuhuhu@hrbeu.edu.cn (S.L.); 993740540@hrbeu.edu.cn (Q.T.); wangliquan@hrbeu.edu.cn (L.W.); dingmx@hrbeu.edu.cn (M.D.); liuzhaojin@hrbeu.edu.cn (Z.L.); 2 Heilongjiang Institute of Technology, College of Mechanical and Electrical Engineering, Harbin 150050, China; 3 College of Shipbuilding Engineering, Harbin Engineering University, Harbin 150001, China; ljw1996@hrbeu.edu.cn (J.L.); qdypy@hrbeu.edu.cn (P.Y.); 923630737@hrbeu.edu.cn (M.X.); pby@hrbeu.edu.cn (B.P.); liukaixin@hrbeu.edu.cn (K.L.)

**Keywords:** hydrodynamic simulation, bionics, attitude adjustment, underwater motion, lift and drag coefficient

## Abstract

Underwater bionic-legged robots encounter significant challenges in attitude, velocity, and positional control due to lift and drag in water current environments, making it difficult to balance operational efficiency with motion stability. This study delves into the hydrodynamic properties of a bionic crab robot’s shell, drawing inspiration from the sea crab’s motion postures. It further refines the robot’s underwater locomotion strategy based on these insights. Initially, the research involved collecting attitude data from crabs during underwater movement through biological observation. Subsequently, hydrodynamic simulations and experimental validations of the bionic shell were conducted, examining the impact of attitude parameters on hydrodynamic performance. The findings reveal that the transverse angle predominantly influences lift and drag. Experiments in a test pool with a crab-like robot, altering transverse angles, demonstrated that increased transverse angles enhance the robot’s underwater walking efficiency, stability, and overall performance.

## 1. Introduction

In the current underwater environment, numerous underwater robots encounter challenges in achieving efficient and stable locomotion. As the robot’s velocity increases, it becomes susceptible to involuntary pitching and sinking, leading to heightened power consumption and difficulties in attitude control. This constitutes a significant obstacle in underwater robot research [1]. Natural biological evolution, spanning millions of years, has resulted in highly adapted creatures with well-suited body structures and motion patterns that enable efficient and stable movement in their natural habitats. Bionics, born from the study of biological structures and behaviors, serves as a source of inspiration for enhancing and designing robots capable of adapting to intricate working environments [2,3].

Research on bionics is primarily divided into structures and materials. In the field of underwater robotics, research related to structural bionics includes bionic fish [4,5], lobsters [6], turtles [7], manta [8], snakes [9], and other animals. The morphology, movement, and propulsion mechanisms of these animals are studied to design robots that are adapted to the underwater environment. Material biomimicry focuses on the biomimetic hierarchical surfaces of aquatic organisms. The physical mechanisms of wetting transitions are studied to design highly stable hydrophobic materials [10]. Wen et al. present the first study of the design, fabrication and hydrodynamic testing of a synthetic, flexible, shark skin membrane [11]. Wu et al. successfully prepared nano SiO_2_ particles modified organic silane coatings aim at the application of the self-cleaning coating on PMMA substrate for deep-sea optical windows [12]. These materials can be used to reduce the water drag of robots.

Crabs have a unique biological structure and form of locomotion due to natural selection and evolution. They inhabit various environments, such as sand, mud, reef, coral, and land–water interface zones, and can move efficiently in all of them [13,14]. Therefore, we selected crabs as bionic objects. Our observation revealed that the crab’s gait varies in different environments. One aspect of crab movement involves adjusting the size and direction of the driving force to change its direction and velocity. Another aspect involves adjusting the crab’s shell posture to cope with water flow disturbance and reduce the effect of lift and drag on its motion due to changes in relative water velocity to the shell. As the crab shell provides most of the lift and drag, the legs primarily serve to adjust the crab shell’s attitude; therefore, the crab shell is the main focus of this research. To enhance the efficiency and stability of the robot in water, this paper examines the crab’s underwater motion attitude and analyzes the impact of the crab shell’s attitude change on lift and drag during underwater motion through simulation and experiment. The data support future work on optimizing bionic shells and planning motion gait for underwater robots to improve their efficiency and stability in water.

Researchers have conducted extensive work on crabs and related bionic studies. Through the observation of crabs, the various forms of movement are summarized as forward walking [15], transverse walking [16], running [17], and bounding [18]. Vidal-Gadea, A. G. et al. observed the range of motion of muscles and joints through anatomical observation. They concluded that the animals tended to have a greater range of joint motion in the preferred direction of movement. The leg segments close to these joints were relatively long, as concluded by [19]. Blake, R. experimentally discussed the effect of flow velocity on the lift-to-drag ratio of crab shells [20]. Through prototype tests, Chen, Y. et al. demonstrated that a crab-type multipedal robot performed better transverse walking than forward walking [21]. Kinugasa, T. et al. discussed that the leg movements of living creatures are highly dependent on the body involved in the dynamics and biological characteristics [22]. Zhang, Jun, et al. investigated the limb structure, kinematics, and gait of a crab using a high-speed camera and pressure sensors and proposed a novel design method for robotic legs [23]. Nguyen, D.N. et al. designed a compliant miniature gripper in a sand blister crab style by observing a crab [24], and so on.

To effectively observe organisms, a more common approach is to observe the trajectories of organisms using direct linear transform (DLT) algorithms and then to conduct relevant studies [25,26,27,28,29]. There are many similar bionic studies. Sheppard, K. A. et al. abstracted the slotted delta wing from the ubiquitous multi-bladed propeller geometry in nature [30]. The team led by Goldman and Chen Li summarized the contact model between footed legs and particle media, as well as the empirical formula in frictional fluids, by studying the movement of lizards in the sand [31,32,33,34,35,36]. Ergin et al. investigated the hydrodynamics and locomotion mechanisms of the Euglena Gracilis (E. Gracilis) using microscopic shadow imaging and microparticle image velocimetry (MicroPIV) [37]. Segall, M. et al. investigated the effect of a snake head shape on the hydrodynamics experienced during trapping [38].

In previous studies, we examined the crab-like robot’s underwater bounding gait [18], swimming paddle design and flapping trajectory [39], and the effects of leg mechanism kinematic modeling and swimming paddle structural parameters on hydrodynamics [40], and demonstrated that the crab-like robot has good mobility capabilities in an amphibious environment. This paper presents a relevant study on the impact of bionic shell attitude change on lift and drag, which is utilized to optimize the underwater motion attitude of the crab-like robot, thereby establishing a research foundation for the promotion and autonomous motion control of crab-like robots. The main contributions of this study are as follows:

1. The hydrodynamic simulation of the robot’s bionic shell is based on attitude data obtained from biological observation. The simulation data are processed to eliminate the effects of parameters such as water velocity, frontal area, and shell shape. The analysis of the processed data focuses on the main attitude parameters of the robot and their impact on lift and drag during underwater locomotion.
2. The experimental platform for the crab-like shell was constructed in a circulating water tank. Force sensors measured the lift and drag of the crab-like shell under different attitude parameters. The simulation data were compared with the processed data, and they exhibited similar values and trends to the experimental data. This suggests that hydrodynamic simulation can yield higher water flow velocity and more significant attitude changes in the future.
3. Underwater pool experiments were conducted using a crab-like robot to validate the improvement in underwater robotic locomotion resulting from adjustments in attitude parameters. The robot performed transverse walking at a consistent speed while varying the transverse angle during the walking process. An analysis was conducted to examine variations in the robot’s underwater locomotion performance by recording data on power consumption and attitude changes at different transverse angles.

Experimental research has demonstrated that the transverse angle of the crab-like robot shell is the primary factor affecting lift and drag during the robot’s motion. By adjusting the transverse angle of the robot during underwater motion, it is possible to effectively enhance both the efficiency and stability of the robot’s movement. Depending on various motion surfaces and operational requirements, suitable lift and drag can be chosen based on hydrodynamic simulation results, thereby selecting the appropriate underwater operational posture for the robot to improve its operational stability and duration. This research methodology can be applied to optimize the motion gaits of other multi-legged biomimetic robots.

This paper analyzes the underwater motion attitude of Portunus trituberculatus and its impact on the lift and drag of the crab shell. The findings are then applied to optimize the underwater walking attitude of a crab-like robot. Section 2 details the construction of a motion observation platform to observe the crab’s underwater walking process. The video is then processed using the DLT algorithm to obtain attitude data. Section 3 simulates the hydrodynamic performance of the robot bionic shell using SATR-CCM+ software. The lift and drag obtained from the simulation are processed dimensionlessly. Then, data analysis software is used to analyze the attitude parameters that affect the lift and drag of the crab-like shell. In Section 4, an experimental setup is designed to verify the accuracy of the hydrodynamic simulation data by conducting experiments in a circulating water tank. This section discusses the selection of lift and drag coefficients for robots based on their motion surfaces and analyzes the underwater motion attitude suitable for different surfaces. Pool experiments were conducted using a crab-like robot to verify the effectiveness of this study in optimizing the robot’s motion gait. Section 5 summarizes the main work of this paper and provides an outlook for subsequent extended work.

## 2. Biological Observation

### 2.1. Biological Selection and Treatment

**Figure 1:**
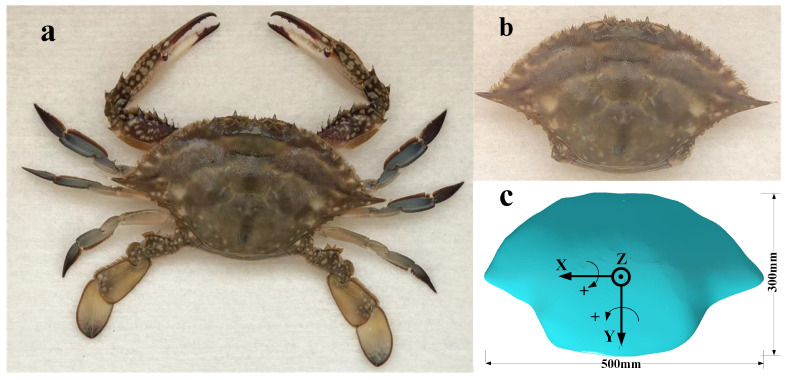
Bionic observation objects. (**a**) Portunus; (**b**) Portunus’ shell part for coordinate measuring machine scanning; (**c**) crab shell model obtained after scanning, with the x-axis positive direction being the forward direction of the crab when it walks horizontally to the left and the z-axis positive direction being the z-axis positive direction of the geodetic coordinate system. (The Portunus’ shell specimens used for scanning in the study were dead crabs purchased from seafood markets in compliance with local regulations and ethical guidelines.)

The chosen crab species is Portunus trituberculatus (Portunus) from the Bohai Sea, as shown in Figure 1a. It is primarily found in shallow offshore waters with depths ranging from 10 to 50 m. The species’ adaptability to its environment is demonstrated by its highest population density being found in muddy and sandy substrates at depths of 10–30 m [41]. Portunus is equipped with a pair of swimming paddles, which enable efficient movement through water and agile crawling on mud and sand. For our observations, we selected 13 crabs of varying sizes to encompass the entire size range of adult crabs in the field. To mitigate errors arising from individual differences, we normalized the movement speed and walking height of the crabs by dividing these measures by their respective body lengths. Additionally, a crab shell, depicted in Figure 1b, was utilized to create a model using a coordinate measuring machine, as shown in Figure 1c, for hydrodynamic simulation.

**Figure 2:**
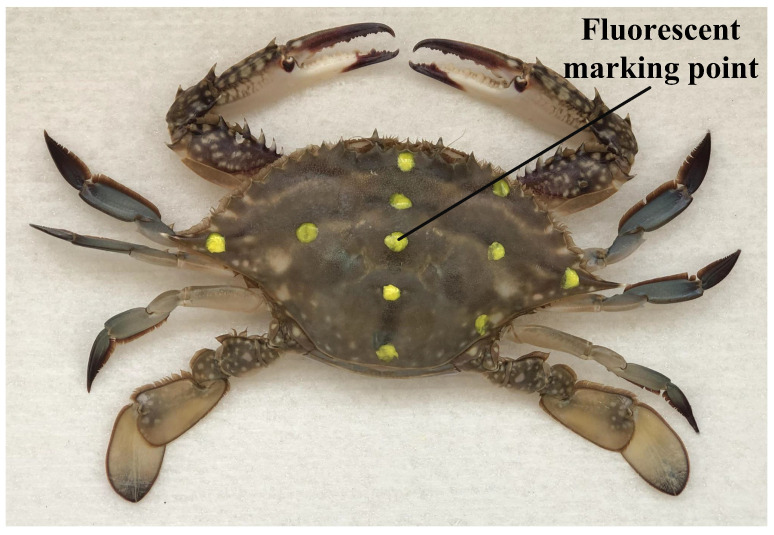
Fluorescent marker distribution.

Figure 2 shows the application of a non-damaging fluorescent pigment to create 6–10 dot markings on the crab shell for precise measurement of crab postures. Recording the crab’s movements involved the use of two high-speed cameras. The DLT algorithm, as detailed by [42], was used to calculate trajectories for the marked points on the crab shells, ultimately yielding data on crab shell attitudes.

### 2.2. Motion Observation System

In order to observe and analyze the movement characteristics of Portunus, a glass aquarium was ordered by the laboratory to construct the movement observation system. The aquarium measured 1500 mm × 600 mm × 600 mm in length, width, and height, with a wall thickness of 15 mm. A seawater recirculation system was installed in the aquarium, with the water temperature controlled at 20 ± 2 °C, the salinity range at 20–23‰, and the pH value range at 7.8–7.9. An oxygenator was used to produce oxygen for the artificial seawater. Gravel and coral stones were placed at the bottom of the tank. The coral stones were separated to prevent the crabs from drilling into them during movement. The observation area was 1000 mm in length.

**Figure 3:**
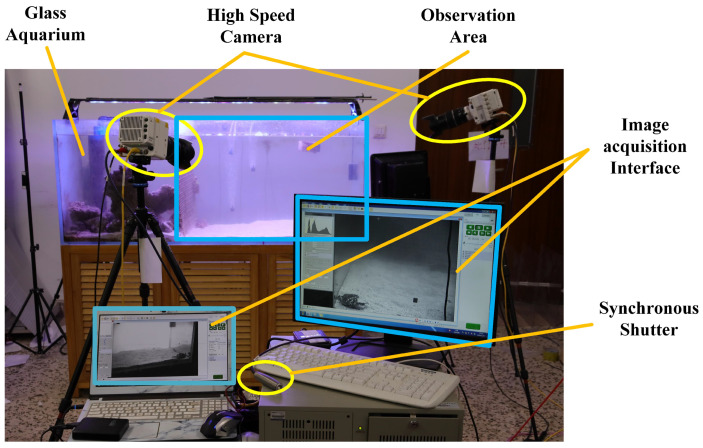
Motion observation system.

Figure 3 shows the motion observation system. Two high-speed cameras (Phantom VEO/VEO4K-L) were arranged at the front left and right sides of the aquarium tank and were triggered to start shooting at the same time by synchronizing their shutters to record the crabs’ movement. Before each shot, the gravel at the bottom of the tank was carefully flattened, and food was used to entice the crabs to walk in a straight line along the tank wall. A total of 200 video sets were recorded, with each recording interval being longer than 15 min to ensure that the crabs were rested. From these recordings, 45 sets of videos with different speeds and postures were selected for data processing. Cases involving swimming, deviation from the trajectory, contact with the tank wall, and obstruction of marking points were excluded to ensure accuracy.

### 2.3. DLT Algorithm Processing Data

**Figure 4:**
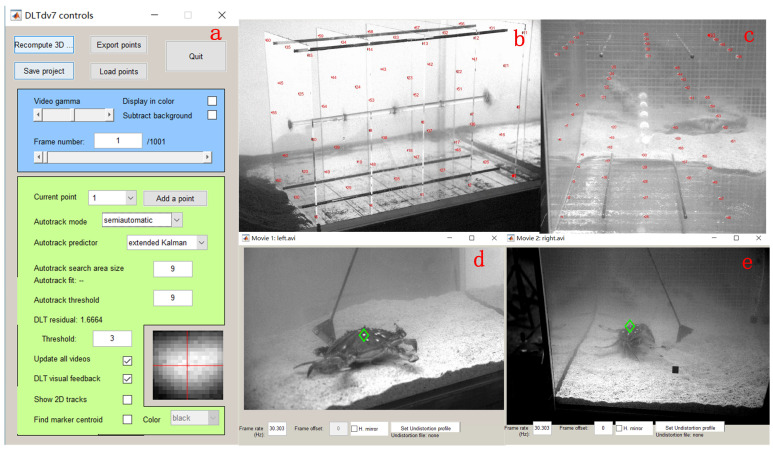
DLT software operation interface and processing. (**a**) DLT software calibration interface; (**b**) front side high-speed camera calibration situation; (**c**) right side high-speed camera calibration situation; (**d**) display of marking points in the front side video (highlighted in the green box in the figure); (**e**) display of marked points in the right side video, with the marked points within the green box in the figure.

The DLTdv toolkit, developed by Tyson L. Hedrick [43], was used to analyze the video data. The software interface, shown in Figure 4a, has two primary functions. The first function calibrates the camera, as illustrated in Figure 4b,c, to derive the transformation matrix between the two cameras using calibration data. The second function involves processing video data to extract three-dimensional information about all marked points during crab movement. Figure 4d,e illustrates the frame-by-frame tracking of marked points during video processing. Video S1 of the supplementary material shows the DLTdv toolkit used to process the crab video and obtain the posture data of the crabs when walking.

### 2.4. Biological Observation Data

**Figure 5:**
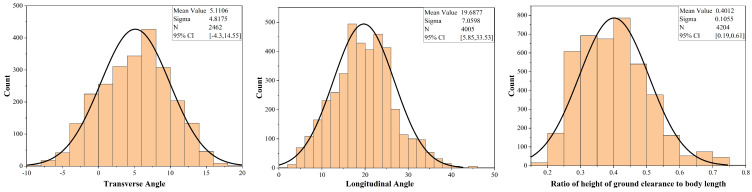
Biological observation data.

The transverse angle of the crab shell (rotated around the y-axis, as shown in Figure 1c), the longitudinal angle (rotated around the x-axis, as shown in Figure 1c), and the height of ground clearance during the crab’s walking can be determined from the 3D marker point information, as depicted in Figure 5. During walking, the crab’s transverse angle, longitudinal angle, and height of ground clearance to body length ratio follow a normal distribution. The relative motion velocity ranges 0–2 times the body length per second, and the movement of the crab is mostly swimming after exceeding this interval. The transverse angle, longitudinal angle, and height of ground clearance/body length were taken as the 95% probability distribution intervals as the common motion attitude parameters of the crab, and the hydrodynamic simulation was carried out to analyze the effect of the crab shell attitude on the lift and drag by selecting the appropriate parameters within this range.

## 3. Crab Shell Hydrodynamic Simulation

### 3.1. Hydrodynamic Flow Field and Grid Setting

To improve the accuracy of the force sensor data during experimental verification and reduce the impact of water flow disturbance and sensor precision, this study proportionally scales up the crab shell model. This adjustment ensures that the lift and drag measured by the force sensor remain within a reasonable range. The size of the crab shell model is set to 500 mm × 300 mm × 160 mm, based on simulation results and the sensor measurement range. Table 1 details the attitude parameters selected for simulation by combining the dimensions of the crab shell model with Figure 5.

**Figure 6:**
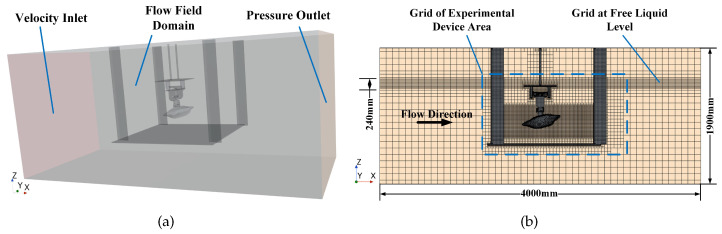
Hydrodynamic simulation flow field and grid setting. (**a**) The range of fluid domain and the arrangement of experimental equipment in the fluid domain; (**b**) the grid setting in the fluid domain.

The STAR-CCM+15.04.010.R8 simulation software was used to conduct a hydrodynamic simulation of the crab shell model. Figure 6a shows the flow field established by the simulation software. The dimensions of the fluid domain were set to 4000 mm × 1700 mm × 1900 mm, which matches the working interval of the circulating water tank test. The experimental device was placed in the fluid domain, and the base mesh size was set to 80 mm. The generated mesh type was a hexahedral mesh. The wall boundaries were designated as follows: the lower wall, the negative x-axis wall as the velocity inlet boundary with an assigned simulated flow rate, and the positive x-axis wall as the pressure outlet boundary. The remaining three walls were designated as symmetric plane boundaries. The experimental device was set as a wall boundary condition, and the distance of the free liquid surface from the lower wall was maintained at 1500 mm.

The mesh was refined around the experimental device in the area of 1650 mm × 800 mm × 1300 mm and around the crab shell model in the area of 1300 mm × 400 mm × 400 mm, with refined mesh sizes of 40 mm and 10 mm, respectively. Additionally, the area 120 mm above and below the free surface of the multiphase flow was encrypted to ensure the accuracy of the wave simulation. The wake region of the crab shell model was refined to improve the accuracy of the flow field conditions, resulting in a more precise lift and drag simulation. Figure 6b shows the final grid effect.

Considering that the highest flow velocity in the circulating water tank during experimental verification was 1.2 m/s, the simulated water flow velocity was set to 1.2 m/s. The main scale of the model is 0.5 m and the flow velocity is 1.2 m/s, then the Reynolds number can be calculated to be about $6.7\times 10^{5}$, the flow is fully developed and considered as turbulent. The unsteady Reynolds-averaged Navier-Stokes equations are chosen as the controlling equations, and the turbulent flow field is simulated using the k-epsilon two-layer model, which is a typical high Reynolds number model based on the full development of turbulence, and is able to accurately solve the force situation of the robot in the water. To enhance the simulation’s accuracy, we utilise Eulerian multiphase flow and establish a VOF hydrostatic wave to replicate the free liquid surface. Video S2 of the supplementary material shows the hydrodynamic simulation of the bionic crab shell model using STAR-CCM+ software.

### 3.2. Dimensionless Treatment of Lift and Drag

To eliminate the effects of crab shell size, media type, and water velocity, we normalized the lift and drag forces to derive dimensionless coefficients. We introduced the lift coefficient $C_{L}$ and drag coefficient $C_{D}$ through standard equations.

(1) $$F_{D}=\frac{1}{2}\rho Sv^{2}C_{D}$$

(2) $$F_{L}=\frac{1}{2}\rho Sv^{2}C_{L}$$

where $F_{L}$ and $F_{D}$ represent the lift and drag forces measured through simulation or experiment, respectively. ρ,*v*,*S* represent the fluid density, flow velocity, and frontal area of the crab shell, respectively.

In this research, changes in the frontal area of the crab shell are caused by variations in the transverse angle. The formula for expressing the frontal area is as follows, given that the transverse angle does not exhibit significant variation:

(3) $$S=\frac{S_{0}}{\cos\theta_{T}}$$

where $S_{0}$ represents the frontal area of the shell on the downstream flow surface when the transverse angle is 0, and $\theta_{T}$ denotes the transverse angle of the crab shell.

Upon incorporating Equation (3) into Equations (1) and (2), we obtain

(4) $$C_{D}=\frac{2F_{D}cos\left(\theta_{T}\right)}{\rho S_{0}v^{2}}$$

(5) $$C_{L}=\frac{2F_{L}cos\left(\theta_{T}\right)}{\rho S_{0}v^{2}}$$

### 3.3. Analysis of Simulation Results

**Figure 7:**
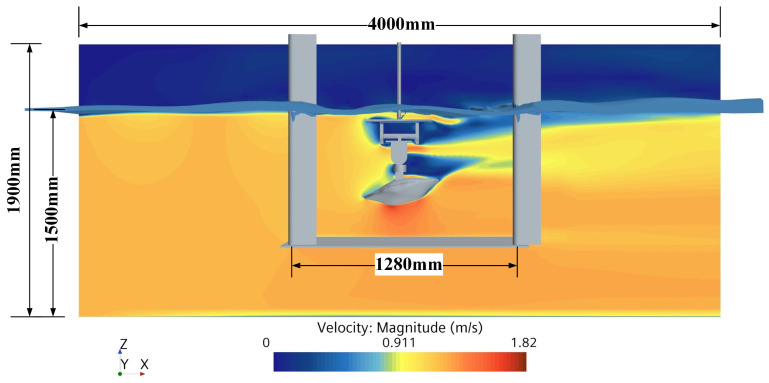
Simulated flow field diagram. The distribution of flow field at 13° transverse angle, 7° longitudinal angle, and 204 mm ground clearance height.

As Figure 7 shows in the flow field diagram of the simulation, a total of 125 attitudes of lift and drag on the crab shell model under the impact of 1.2 m/s current velocity were simulated with reference to the attitude parameters in Table 1. The simulated lift and drag of the crab shell model were then transformed from the geodetic coordinate system to the model coordinate system, and the lift and drag coefficients were calculated. The lift and drag coefficients were analyzed using Minitab 20.3 software, employing correlation, optimal subset, and multiple regression techniques.

Based on the lift and drag data obtained from the simulation for 125 sets of attitudes, the *p*-Values for the relationships between the independent variables, transverse angle (TA), longitudinal angle (LA), and height of ground clearance (H), and the dependent variables, lift coefficient ($C_{L}$) and drag coefficient ($C_{D}$), are all less than 0.01, which is statistically significant.

**Figure 8:**
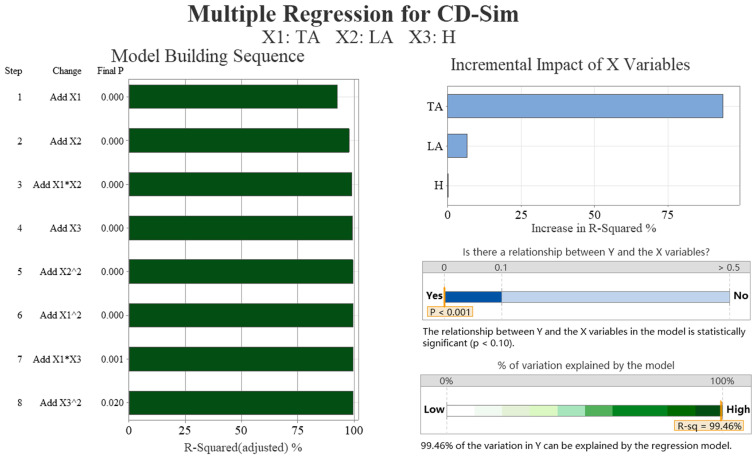
The multiple regression analysis results of the drag coefficient of simulation data ($C_{D}$-Sim). We conducted data analysis on four aspects: significance analysis of samples, regression model error, model construction order, and the proportion of independent variable increment impact.

Table 2 shows a strong negative correlation between the drag coefficient obtained from the simulation and the transverse angle (−0.961) and weak correlations with the longitudinal angle (0.229). There is no correlation with the height of ground clearance, which is not statistically significant. Table 3 outlines the optimal subsets for the drag coefficients, which include the transverse and longitudinal angles. The addition of the variable height of ground clearance did not result in a significant reduction in the Mallows’ Cp values. The results of the multiple regression analysis are presented in Figure 8. The figure shows a mainly linear relationship between the drag coefficient and the transverse and longitudinal angles. In particular, the transverse angle has a significant impact on the drag coefficient, accounting for over 75% of the influence. The R-squared value of 99.46% indicates that the regression model’s drag coefficient is highly consistent with the simulation data.

**Figure 9:**
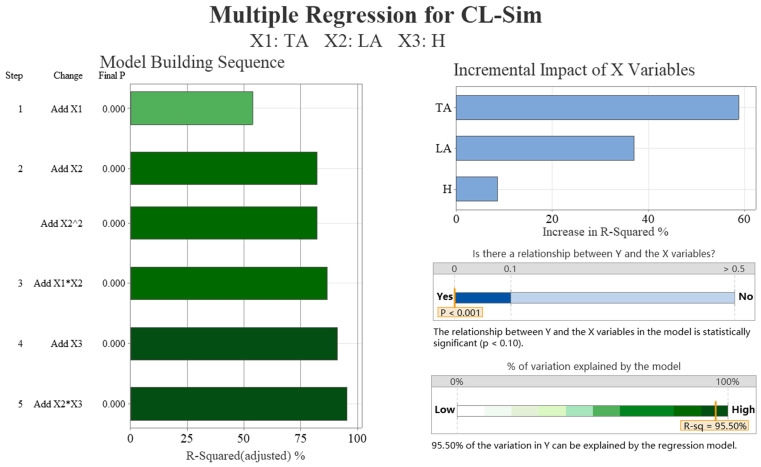
The multiple regression analysis results of the lift coefficient of simulation data ($C_{L}$-Sim). We conducted data analysis on four aspects: significance analysis of samples, regression model error, model construction order, and the proportion of independent variable increment impact.

Table 4 shows a strong negative correlation (−0.736) between the lift coefficient obtained from the simulation and the transverse angle and weak correlations with the height of ground clearance (−0.210) and longitudinal angle (−0.261). Table 5 presents the optimal subsets for the lift coefficients, which include the transverse and longitudinal angles. The addition of the variable height of ground clearance did not result in a significant reduction in the Mallows’ Cp values. Figure 9 presents the results of the multiple regression analysis. The figure illustrates a primarily linear relationship between the lift coefficient and the transverse and longitudinal angles. The combination of these two angles has a significant impact on the lift coefficient, accounting for over 75% of the effect. Additionally, the height of ground clearance and cross-term items have some effects. The R-squared value of 95.50% indicates that the regression model’s lift coefficient is highly consistent with the simulation data.

In summary, the transverse angle is the primary factor that influences the drag coefficient of the simulation data ($C_{D}$-Sim) of the crab shell model, with a predominantly linear relationship with the drag coefficient. The longitudinal angle has a minor and linear impact on $C_{D}$-Sim, while changes in the height of ground clearance do not affect $C_{D}$-Sim. The lift coefficient of simulation data ($C_{L}$-Sim) of the crab shell model is primarily influenced by the transverse and longitudinal angles, with their impact being primarily linear. However, the longitudinal angle has a lesser effect on $C_{L}$-Sim compared to the transverse angle, and changes in height of ground clearance exhibit a smaller impact on $C_{L}$-Sim.

**Figure 10:**
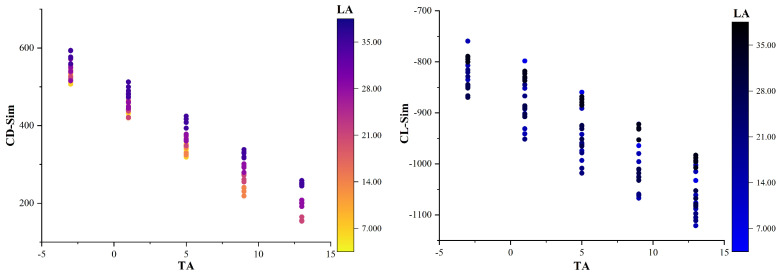
Scatter plots of $C_{D}$-Sim and $C_{L}$-Sim with changes in transverse angle (TA) and longitudinal angle (LA).

To observe changing trends more clearly, Figure 10 presents scatter plots of $C_{D}$-Sim and $C_{L}$-Sim with changes in transverse angles (TAs) and longitudinal angles (LAs). $C_{D}$-Sim decreases as the transverse angle increases and increases as the longitudinal angle increases. Likewise, $C_{L}$-Sim decreases as the transverse angle increases. The impact of factors such as height of ground clearance (H), TA × LA, and H × LA on $C_{L}$-Sim is not intuitive. This is in line with the correlation and regression model analyses.

The robot’s stability during water walking is enhanced by the negative lift coefficient, which reduces the influence of buoyancy. Additionally, the decrease in the drag coefficient results in less water flow drag when the robot moves in water. This reduces the power consumption required to overcome drag and increases the efficiency of the robot’s movement. The simulation results show that increasing the transverse angle of the robot during underwater walking improves its motion efficiency and stability.

The phenomenon known as the wall effect can be used to explain the situation where the lift coefficient is negative and is influenced by the height of the ground clearance, as described by the Bernoulli principle in Equation (6).

(6) $$\frac{1}{2}\rho v^{2}+p+\rho gz=const$$

where ρ is the density of the liquid, *v* is the relative velocity of the water flow to the model, *p* is the pressure at that point, and *z* is the height at that point.

A lower height of ground clearance causes greater water flow velocity on the lower surface of the model and lower pressure at that location. This results in a downward pressure on the entire model, leading to a negative lift coefficient. Conversely, increasing the height of ground clearance weakens the wall effect, creating a reduced influence on the lift coefficient.

## 4. Experiment

### 4.1. Circulating Water Tank Experiment

To verify the hydrodynamic simulation results, we designed an experimental platform in a circulating water tank to test the crab shell model. The tank is 7 m long, 1.7 m wide, and 1.5 m deep, providing a flow field environment of 0.1–1.2 m/s. Due to the minimum error in water flow velocity in the middle depth area of the circulating water tank, we designed a simulated bottom device at a depth of 0.75 m to simulate the bottom surface. The experimental data were obtained using ATI’s Gamma IP 68 force sensor. The sensor parameters are detailed in Table 6. The experimental shell was fabricated using a 3D printer to ensure conformity with the dimensions of the simulated model.

To obtain lift and drag information of the crab shell model in different attitudes, it is necessary to design the experimental setup in conjunction with the circulating water tank due to limitations of the experimental site and environment. When designing the experimental setup, we try to minimise the influence of the experimental setup and the site on the flow field of the crab shell, such as increasing the distance of the support columns as much as possible, wrapping the teardrop-shaped shells on the surface of the support columns to guide the flow, and adding a simulated bottom device.

**Figure 11:**
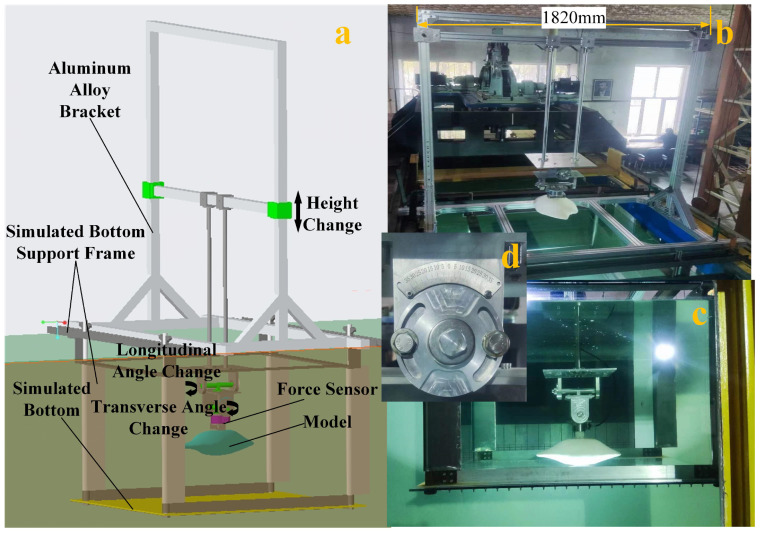
The experimental verification platform. (**a**) The three-dimensional model of the experimental platform. (**b**) The aluminum alloy support frame installed and fixed on the circulating water tank, where the ground height of model can be adjusted. (**c**) The simulated bottom and model parts in the circulating water tank. (**d**) The transverse and longitudinal angle adjustment devices. The dial can be adjusted to the appropriate angle, and the nut tightened to secure it.

**Figure 12:**
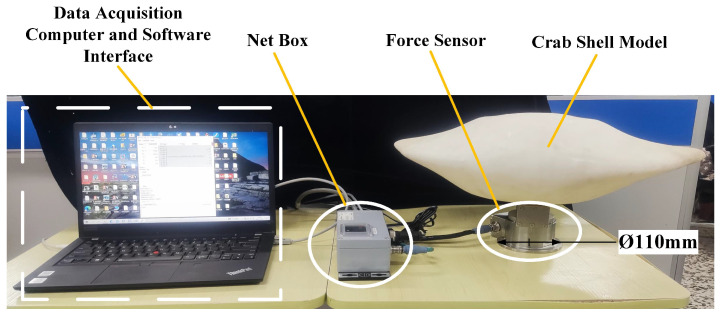
The sensor acquisition device.

Figure 11a shows the design of the experimental platform, which includes a simulated bottom, support frame, attitude parameter adjustment device, sensor acquisition device, and other apparatus. The primary components of the experimental platform are the simulated bottom and an aluminum alloy bracket. Four support columns connect the simulated bottom to the acrylic plate and channel steel. Nuts can be used to adjust the length of each support column, ensuring parallel alignment of the simulated bottom with the circulating water tank. To minimize disruption to the flow field, the support column is shaped like a water drop, guiding water flow around it. Figure 11b–d displays the experimental platform deployment in a circulating water tank. The platform comprises the above-water section, underwater section, and attitude parameter adjustment device. The attitude parameter adjustment device can adjust the transverse angle, longitudinal angle, and height of ground clearance of the model in water. To eliminate the influence of crab shell buoyancy on measurements, the sensor data are reset to zero in stable water. Data collection commences only after the water flow rate stabilizes. The sensor acquisition device is depicted in Figure 12 and comprises a computer, a digital acquisition device, and a sensor. File S1 of the supplementary material shows the 3D model file of the experimental platform is shown in the Supplementary Material’s, which contains components such as the circulating water tank, support frame, simulated bottom, attitude parameters adjustment device, the sensors, and the model.

Using the attitude parameter data presented in Table 1, we measured the lift and drag acting on the model for 125 sets of shell attitudes in a water flow velocity field of 1.2 m/s. Video S3 of the supplementary material shows an experiment with a crab shell model in a circulating water tank to obtain data on the lift and drag of the model at different water velocities.

### 4.2. Comparison of Experimental and Simulation Results of the Crab Shell Model

The crab shell model’s height of ground clearance may vary depending on adjustments made to the transverse and longitudinal angles, which are determined by the experimental equipment’s structural design. Before conducting data analysis, it is crucial to calculate the height of ground clearance by integrating the transverse and longitudinal angles with the experimental platform structure. Similar to the analysis of simulation data, the lift and drag coefficients in the experimental data should be calculated based on the provided experimental data. To examine the influencing factors on the lift and drag coefficients, Minitab20.3 software was used to conduct a correlation analysis, an optimal subset analysis, and multiple regression analysis.

**Figure 13:**
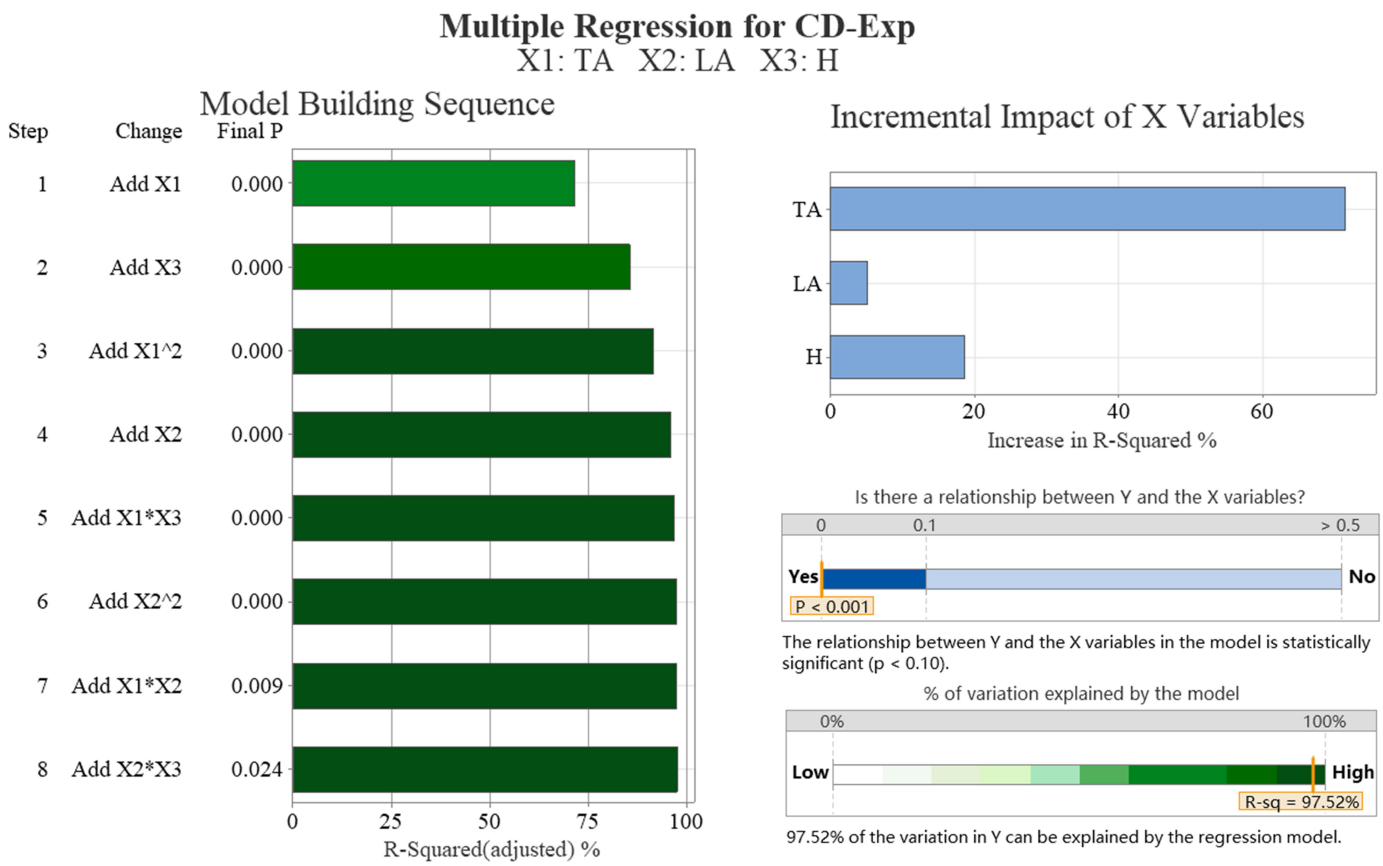
The multiple regression analysis results of the drag coefficient of experimental data ($C_{D}$-Exp). We conducted data analysis on four aspects: significance analysis of samples, regression model error, model construction order, and the proportion of independent variable increment impact.

Table 7 shows a negative correlation (−0.847) between the drag coefficient obtained from the experiment and the transverse angle. However, there is a weaker negative correlation (−0.435) with the height of ground clearance and no correlation with the longitudinal angle, which is not statistically significant. The optimal subset of drag coefficients, as shown in Table 8, includes the transverse angle and height of ground clearance. Figure 13 shows the results of the multiple regression analysis, indicating a linear relationship between the drag coefficient and the transverse angle. The transverse angle has a significant impact on the drag coefficient, accounting for nearly 75%. The R-squared value of 97.52% indicates that the regression model’s drag coefficient is highly consistent with the experimental data.

**Figure 14:**
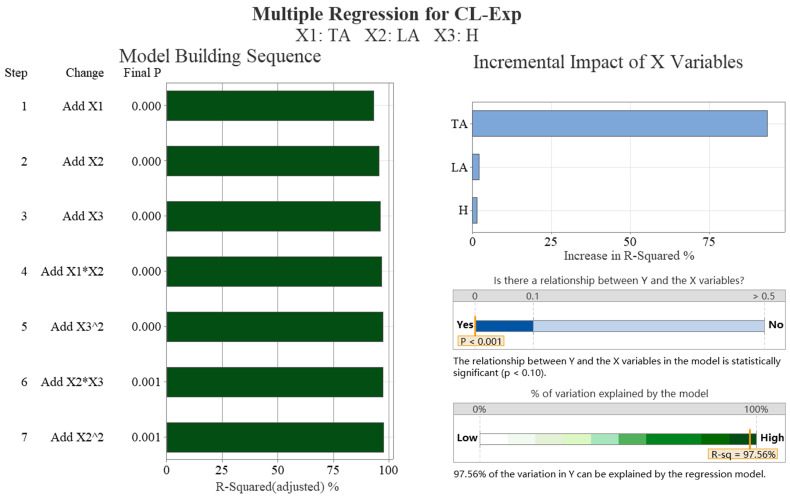
The multiple regression analysis results of the lift coefficient of experimental data ($C_{L}$-Exp). We conducted data analysis on four aspects: significance analysis of samples, regression model error, model construction order, and the proportion of independent variable increment impact.

Table 9 shows a strong negative correlation (−0.964) between the lift coefficient obtained from the experiment and the transverse angle. However, there is only a weak positive correlation (0.153) with the longitudinal angle and no correlation with the height of ground clearance, which is not statistically significant. The optimal subsets of lift coefficients, as shown in Table 10, include the transverse and longitudinal angles. Figure 14 presents the results of the multiple regression analysis. The illustration shows that the drag coefficient has a linear relationship with both the transverse and longitudinal angles. The impact of the transverse angle on the lift coefficient is significantly greater than 75%. The R-squared value of 97.56% indicates that the regression model’s drag coefficient is highly consistent with the experimental data.

The experimental data show that the drag coefficient is mainly correlated with the transverse angle and height of ground clearance, while the lift coefficient is mainly associated with both the transverse and longitudinal angles. The transverse angle is the most influential factor, affecting both lift and drag coefficients and contributing to over 75% of the overall impact. The simulation and experimental data consistently show R-squared values exceeding 95%, which supports the effectiveness of fitting appropriate regression models using the transverse angle, longitudinal angle, and height of ground clearance.

The height of ground clearance has a significant impact on the drag coefficient during the experimental data, unlike the simulation data. It is evident from a thorough analysis of both the experiment and the data processing procedures that the structural issues with the experimental equipment mentioned at the beginning of this section are the most likely cause of this situation. Variations in the height of ground clearance can be induced by adjusting the longitudinal angle. Table 7 and Table 9 show a correlation of 0.389 between the height of ground clearance and the longitudinal angle, indicating that the independent variables are not entirely independent. This lack of independence can introduce errors in the data analysis process.

**Figure 15:**
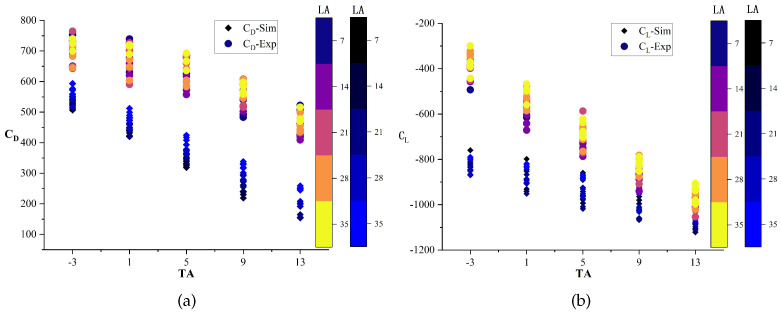
Comparison diagram between simulation and experimental data. (**a**) The drag coefficient varies with the transverse angle (TA) and longitudinal angle (LA). The color of the points in the figure represents the variation in the longitudinal angle. The dots represent experimental data, with colors ranging from black to blue. The diamond point simulation data shows colors ranging from purple to yellow. (**b**) The variation in lift coefficient with transverse and longitudinal angles.

Figure 15 shows the variation of drag and lift coefficients in experimental and simulation models at transverse and longitudinal angles. This allows for a more intuitive observation of the data. The figure illustrates a primarily linear relationship between $C_{D}$-Exp and transverse angle (TA), which decreases as the transverse angle increases, indicating a negative correlation. Additionally, there is a positive correlation between $C_{D}$-Exp and longitudinal angle (LA), which increases as the longitudinal angle increases. However, this correlation is not significant enough to be apparent from the figure. Similarly, $C_{L}$-Exp exhibits a mainly linear relationship with transverse angle (TA), which decreases as the transverse angle increases, indicating a negative correlation. The experimental results indicate a positive correlation between $C_{L}$-Exp and the longitudinal angle (LA) increase, which is consistent with the previous correlation analysis.

Despite some numerical differences, the trends of the lift and drag coefficients for both simulation and experiment are consistent. The lift coefficient has a larger error in the amount of change between experimental and simulation data than the drag coefficient due to the error brought by the test device on the height above ground and longitudinal angle. Overall, the simulation data can effectively predict the trends of the lift and drag forces in the model. Subsequent studies can use simulation software to calculate the lift and drag coefficients for each robot pose. Additionally, integrating other sensors into the robot control process can help evaluate and predict the forces applied to the robot during motion, improving the stability and efficiency of its movement in an amphibious environment.

The aim of this section is to confirm the simulation’s consistency with the actual experimental results. Due to limitations imposed by the experimental environment and device structure, it is not possible to obtain the force situation of the model in all attitudes and velocity of the flow field. At this stage, simulation can be used to derive the force change rule of the robot crab shell model, which can then guide the adjustment of the robot’s underwater motion attitude. This analysis method also has limitations. For example, when the robot changes its flow field when it changes its motion scenario, it is necessary to re-model the simulated scenario to obtain a more accurate pattern of force changes. For the simulation of complex flow field is also another difficulty of this analysis method, such as the irregular fluctuation of waves, the need for more in-depth study of the simulation method. The process of simulation requires high computer performance and repeated iterations until convergence. Additionally, simulations typically require more time and cost compared to experiments.

### 4.3. Inspiration of Lift and Drag Coefficients on Underwater Attitude Control of Robots

The research content of this paper can be applied to robot motion control. By modelling and analysing the environment and water flow in different motion scenes, we can obtain the change rule of the lift and resistance coefficient of the robot in water. This will enable us to select the appropriate robot motion attitude and achieve effective underwater motion.

An analysis of underwater robot locomotion revealed two distinct types: movement on hard surfaces, such as floors and stones, and movement through particulate media, such as sand and mud.

When the robot is on hard ground, there is no need to consider a scenario where the body becomes trapped in the substrate. In this situation, the robot experiences gravity, buoyancy, and lift in the vertical direction, while drag and friction affect it in the horizontal direction, as shown in Equation (7).

(7) $$\left\{\begin{array}{c} F_{z}=mg-F_{b}-F_{L}\\ F_{x}=F_{f}-F_{D} \end{array}\right.$$

where $F_{z}$ denotes the force acting along the z-axis in the robot coordinate system, $F_{b}$ signifies the buoyancy force experienced by the robot in water, $F_{L}$ represents the lift generated by the impact of water flow on the robot during its movement, $F_{x}$ indicates the force along the x-axis in the robot coordinate system, with the positive direction aligning with the forward movement of the robot, $F_{D}$ accounts for the drag produced by the impact of water flow on the robot during its motion, and $F_{f}$ characterizes the frictional force exerted on the robot, specifically on hard ground, involving dynamic friction.

By expanding $F_{D}$, $F_{L}$, $F_{b}$, and $F_{f}$ and incorporating them into Equation (7), the resulting equation is as follows:

(8) $$\left\{\begin{array}{c} F_{z}=mg-\rho gV-\frac{\rho S_{0}v^{2}C_{L}}{2\cos\left(\theta_{T}\right)}\\ F_{x}=\mu mg-\frac{\rho S_{0}v^{2}C_{D}}{2\cos\left(\theta_{T}\right)} \end{array}\right.$$

Following the preceding text, a negative lift coefficient is observed when the robot’s height of ground clearance is low. This results in increased pressure on the robot’s ground when the relative velocity of the water flow is increased, leading to greater stability in its movement. A smaller magnitude of the negative lift coefficient indicates a more favorable underwater walking posture. As the relative velocity of the water flow increases, the force exerted on the robot $F_{D}$ also increases, causing a corresponding decrease in $F_{x}$. When $F_{x}$ reaches zero, it means that the robot cannot move forward or withstand the impact of the water flow.

(9) $$v_{move}=\sqrt{\frac{2\mu mgcos\theta_{T}}{\rho S_{0}C_{D}}}$$

$V_{move}$ represents the maximum theoretical relative motion velocity at which an underwater robot can propel itself. Robots exceeding this velocity will be displaced by water flow. The drag coefficient’s smaller value results in a higher theoretical maximum velocity, which aligns more favorably with the robot’s motion posture during underwater locomotion.

When operating on a wet particle medium, the robot must consider the potential risk of becoming trapped. The depth of immersion directly affects the friction force encountered. In cases where the lift coefficient is negative, the robot will exert more pressure on the ground as the relative velocity of the water flow increases, increasing the likelihood of becoming stuck in sediment. To calculate the lift and drag coefficients, we used the wet particle medium theory for a simplified analysis, as introduced by Ma et al. [44]. This theory examines the force dynamics when a moving object infiltrates a wet particle medium, as represented by Equation (10).

(10) $$F_{x,z}=\int_{S}\alpha_{x,z}^{v}\left(\beta_{S},\gamma_{S}\right){\left|z\right|}_{S}\epsilon_{v}dA_{S}$$

where $\alpha_{x,z}^{v}\left(\beta_{S},\gamma_{S}\right)$ is the angle of attack $\beta_{S}$, the intrusion angle $\gamma_{S}$, and the stress per unit depth when the velocity is *v*. ${\left|z\right|}_{S}$ is the intrusion depth, $\epsilon_{v}$ is the velocity coefficient, A is the frontal area of the intrusion part, and $F_{x,z}$ is the vertical and horizontal forces.

The depth of a body trapped in a granular medium can be determined by analyzing Equation (10), which shows a direct relationship between the depth and two factors: the vertical force applied and the area of the body’s contact with the granular medium. We can establish a system of equations by providing the vertical and horizontal forces.


{(11)Fz=mg−ρgV−ρS0v2CL2cosθT(12)Fz=∫AαzvβΔA,γΔAHϵvdΔA(13)Ff=∫SαzvβSΔS,γΔSHϵvdΔS(14)Fx=Ff−ρS0v2CL2cosθT

A is the frontal area of the robot invading the particle medium, and S is the projected area of the robot invading the particle medium in the direction of motion perpendicular to the plane. Firstly, Equation (11) can be brought into Equation (12) to calculate the plunging depth H of the robot at this time. Secondly, H can be brought into Equation (13) to calculate the drag of the granular medium, i.e., the friction force $F_{f}$ that the robot is subjected to at this plunging depth. And finally, the horizontal-direction force $F_{x}$ that the robot is subjected to can be calculated by bringing $F_{f}$ into Equation (14). The process undergoes dynamic changes with the relative motion velocity *v*. When $C_{L}$ is negative, a smaller $C_{L}$ results in a greater vertical force $F_{z}$ acting on the robot, leading to an increased frictional force $F_{f}$ experienced by the robot after being trapped in a granular medium. Within the robot’s driving force range, there can be a larger theoretical maximum velocity. The water flow velocity that the robot can withstand is also greater. We can determine the appropriate motion posture and its corresponding $C_{L}$ and $C_{D}$ based on the actual operation situation and optimize the underwater walking posture of the robot accordingly.

Based on the above analyses, it is evident that changes in the motion substrate of the robot result in corresponding changes in the applied forces during its motion. Simulation methods can be employed to study the robot’s behavior in different water flow environments and obtain the lift and drag coefficients for various attitudes of the robot. Thus, we can give the robot with an initial attitude that is close to the optimal solution, we can enhance the efficiency of robot learning and training, and facilitate robot motion control.

### 4.4. Robot Underwater Walking Experiment

To evaluate the improved motion capabilities of the underwater robot discussed in this research, we conducted underwater walking experiments in a water tank. The crab-like robot is 0.66 m long, 0.49 m wide, and 0.38 m high, maximum underwater travelling speed of 0.15 m/s. It weighs 6.5 kg, with a land load of 5 kg and a buoyancy of 42 N. The robot has six walking feet and two swimming feet. It has two modes of operation: remote-controlled and autonomous. It can be switched between three modes of walking on land, walking underwater, and swimming underwater. The robot is capable of horizontal walking, vertical walking, turning in place, and jumping. The robot is powered by SAVOX SW-1210SG servo-steering gear, which has a torque of 32 kg · cm, a speed of 0.13 $s/60^{\circ }$, and an IP67 waterproof rating.

**Figure 16:**
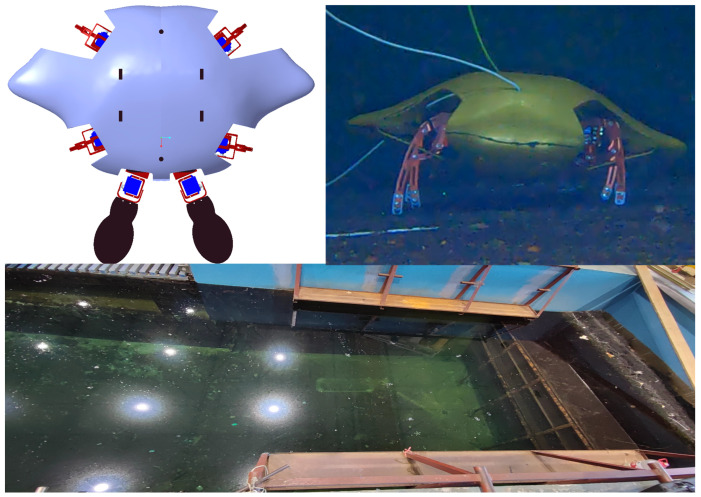
Robot models and experimental sites.

The crab-like robot’s bionic shell was designed based on the simulation model’s shape, measuring 800 mm in length, 460 mm in width, and 320 mm in height. It was 3D printed using photosensitive resin material with a printing accuracy of 0.08–0.1 mm. The hydrophobic material sprayed on the printed bionic shell was a fluorosilane polymer with a solvent of butyl acetate and a specific gravity of 0.8 g/mL. The hydrophobic material had a water contact angle of more than $160^{\circ }$, and a rolling angle of less than $5^{\circ }$. The bionic shell weighs 2.55 kg and has a buoyancy of 22.15 Nm. To regulate the buoyancy of the robot, 1 kg of lead was added to the robot. Figure 16 shows the 3D model of the robot, the underwater walking attitude and the experimental site.

The robot’s control system comprises of the TX2, power module, servo control board, camera, and sensors. Communication with the host computer is achieved through the TCP/IP protocol. Upon receiving the control signal, the robot sends a PWM signal to the servo control board via the TX2 in TTL protocol. The servo control board receives the PWM signal to control the servo and execute the motion commands. The camera is connected to the TX2 via USB to perform target recognition and tracking tasks. The robot’s sensors, including inertial and current sensors, are connected to the TX2 via TTL protocol to provide and record parameters such as attitude changes and power consumption during robot motion.

The simulation and experimental results indicate that the transverse angle is the most significant factor affecting the lift coefficient and drag coefficient of the robot, accounting for over 75% of the total. The impact of longitudinal angle on the lift and drag coefficients is smaller. During underwater walking experiments, the robot’s relative water speed is also low, resulting in an insignificant effect on the robot’s underwater walking when the longitudinal angle is altered. In the experiment on underwater robot locomotion, the transverse angle was used as a variable. The robot was controlled to walk sideways at the same speed with different transverse angles. The change in robot attitude angle and power consumption were recorded during the walking process to analyze the influence of the change in robot attitude on the robot’s underwater movement. Video S4 of the supplementary shows the robot walks in the pool with the bionic crab shell at a given transverse angle.

**Figure 17:**
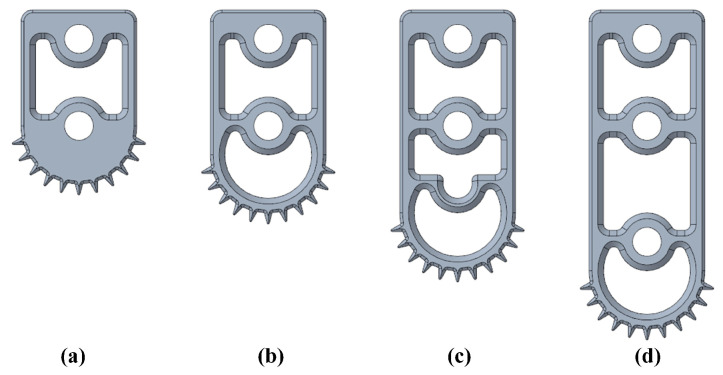
Four different lengths of toes. (**a**) is the toe of the foot after a 5 mm decrease in length, (**b**) is the usual toe length of the robot, (**c**) is the toe of the foot after a 10 mm increase, (**d**) is the toe of the foot after a 20 mm increase.

The robot’s walking leg has limitations in its parallel structure, resulting in a relatively restricted range of height variation for its toe points. To expand the transverse angle variation capabilities of the robot, we modified its gait to maintain a specific height difference between the left and right sides during transverse walking. We created three distinct motion gaits, namely Gait1, Gait2, and Gait3, based on height differences of 0 mm, 15 mm, and 30 mm on the left and right sides. By adjusting the lengths of the left and right walking toes, as shown in Figure 17, we achieved the desired effect of altering the robot’s larger transverse angle. The robot was manipulated to walk laterally within the water tank, and we recorded data from its current sensor and inertial module.

**Figure 18:**
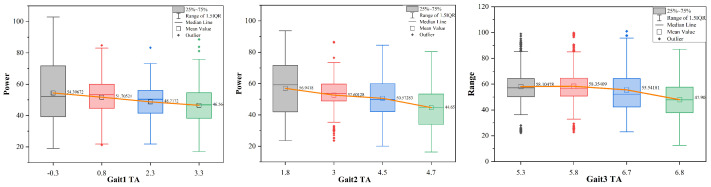
Changes in transverse angle (TA) and average power under three different gaits.

Figure 18 illustrates the changes in transverse angle and average power during three gait movements. The results show that, as the robot maintains a consistent velocity in water under the gait sequences of Gait1, Gait2, and Gait3, there is a significant reduction in power consumption with an increasing transverse angle. Specifically, the average power consumption during Gait1 decreased from 54.4 W to 46.6 W, in Gait2 from 56.9 W to 44.7 W, and in Gait3 from 58.1 W to 47.9 W. Notably, the average power consumption decreased by 15%, 18%, and 21% during the three respective gait movements.

**Figure 19:**
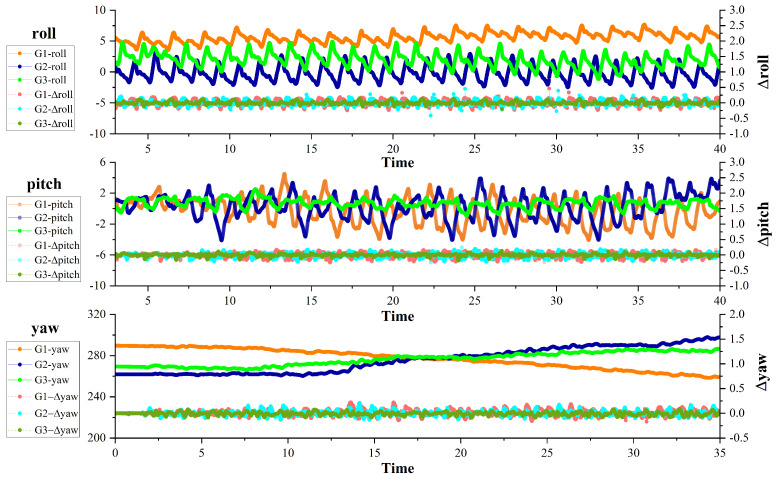
The variation in attitude angle and attitude angle change rate over time during robot motion. G1, G2, and G3 in the figure represent Gait1, Gait2, and Gait3, the three gaits we designed.

Figure 19 shows the temporal variation in the robot’s attitude angle and attitude angle change rate while maintaining a constant velocity across three distinct gaits. The results indicate that, when using Gait3, the robot exhibits significantly smaller changes in roll, pitch, and yaw attitude angles compared to the other two gaits. It is worth noting that Gait3, as per the gait configuration, has the maximum transverse angle during the gait process. The increase in the transverse angle during the robot’s motion reduces power consumption significantly. As a result, the body experiences less change in its various attitude angles during movement. In summary, adjusting posture parameters during the crab-like robot’s underwater motion is crucial for improving both efficiency and stability, improving its underwater movement effect.

## 5. Conclusions and Future Work

The crab’s morphological structure and movement in the amphibious environment make it an excellent predator, deserving of research. Its unique shell shape and adaptive response to water currents optimize movement efficiency and stability while traversing through water. This has significant implications for the development of highly maneuverable underwater robots. This study uses high-speed camera footage to observe and analyze the locomotion of crabs underwater. The crab shell attitude change interval is obtained, and hydrodynamic simulations and experiments are conducted on crab shells to determine the effect of crab shell attitude changes on lift and drag. Lift and drag coefficients are derived using dimensionless processing, thereby reducing the impact of current velocity, density, and the frontal area on lift and drag. Additionally, we verified the effectiveness of our study through robot experiments, thus confirming the reliability of our results. Therefore, the conclusions are as follows:

1. The lift and drag of the bionic shell of the robot are mainly influenced by factors such as relative motion velocity, frontal area, and lift and drag coefficients. The shape of the shell and the underwater motion posture play a crucial role in determining the lift and drag coefficients, with the transverse angle being the primary driver of variations in these coefficients.
2. The relationship between the drag coefficient and the transverse angle is linear, with the drag coefficient decreasing as the transverse angle increases. Similarly, the lift coefficient is negative and also exhibits a linear relationship with the transverse angle, decreasing as the angle increases.
3. The use of hydrodynamic simulation methods for the robot shell allows for the precise determination of lift and drag coefficients in various postures. By analyzing the substrate of the robot’s underwater working environment, appropriate optimization tendencies for $C_{L}$ and $C_{D}$ were selected for different substrates to optimize the robot’s underwater movement.
4. Experimental data from robot trials show that increasing the transverse angle during underwater motion reduces the power expended by the robot in Gait1, Gait2, and Gait3 movements by 15%, 18%, and 21%, respectively, while also improving stability.

This paper presents an optimized and improved method for controlling the walking attitude of a crab-like robot. The study observed the motion characteristics of the sea crab and analyzed the hydrodynamic performance of the bionic crab’s shell in different attitudes. The proposed method was experimentally verified, resulting in reduced power consumption and improved stability of the underwater robot’s operation. This research is significant for controlling the attitude of other multi-legged biomimetic robots.

For future work, it is recommended to expand the range of attitude parameters for simulation by selecting a wider range of transverse and longitudinal angles. This will allow for a more comprehensive analysis of the influence of attitude parameters on lift and drag. Biological observations have revealed that the periphery of the crab shell is covered with flagella, and the surface of the shell is not smooth and has a mucous membrane. Experiments can be designed to investigate the effect of flagella and pits on the hydrodynamic properties of crab shells to optimize the structure of the robotic bionic shell. Mucous film properties on crab shells can also be tested to find suitable hydrophobic coatings to improve the surface properties of crab shell models. When a crab walks in water, its swimming paddles move regularly. The coupling phenomenon between the crab’s walking feet and swimming paddles can be studied to improve the gait of underwater robots.

## Figures and Tables

**Table 1:** Simulation selected attitude parameters.

Parameters	Values				
Transverse angle	$-3^{\circ }$	$1^{\circ }$	$5^{\circ }$	$9^{\circ }$	$13^{\circ }$
Longitudinal angle	$7^{\circ }$	$14^{\circ }$	$21^{\circ }$	$28^{\circ }$	$35^{\circ }$
Ratio of height to body length	0.25	0.33	0.41	0.49	0.57
Height of ground clearance	124 mm	164 mm	204 mm	244 mm	284 mm

**Table 2:** Correlation analysis on drag coefficients in simulation data ($C_{D}$-Sim).

Sample 1	Sample 2	N	Correlation	*p*-Value
H	$C_{D}$-Sim	125	−0.050	0.580
TA	$C_{D}$-Sim	125	−0.961	0.000
LA	$C_{D}$-Sim	125	0.229	0.010
TA	H	125	0.000	1.000
LA	H	125	0.000	1.000
LA	TA	125	0.000	1.000

**Table 3:** Optimal subset of drag coefficient from simulation data ($C_{D}$-Sim).

Vars	R-Sq	R-Sq(adj)	R-Sq(pred)	Mallows’ CP	H	TA	LA
1	92.3	92.3	92.1	308.8		X	
1	5.3	4.5	2.1	5194.4			X
1	0.2	0.0	0.0	5475.3	X		
2	97.6	97.6	97.4	16.0		X	X
2	92.6	92.5	92.2	296.9	X	X	
2	5.5	4.0	0.7	5182.4	X		X
3	97.8	97.8	97.7	4.0	X	X	X

**Table 4:** Correlation analysis on lift coefficients in simulation data ($C_{L}$-Sim).

Sample 1	Sample 2	N	Correlation	*p*-Value
H	$C_{L}$-Sim	125	−0.210	0.019
TA	$C_{L}$-Sim	125	−0.736	0.000
LA	$C_{L}$-Sim	125	−0.261	0.003
TA	H	125	0.000	1.000
LA	H	125	0.000	1.000
LA	TA	125	0.000	1.000

**Table 5:** Optimal subset of lift coefficient from simulation data ($C_{L}$-Sim).

Vars	R-Sq	R-Sq(adj)	R-Sq(pred)	Mallows’ CP	H	TA	LA
1	54.1	53.7	52.6	39.2		X	
1	6.8	6.1	3.4	204.3			X
1	4.4	3.6	1.2	212.8	X		
2	60.9	60.3	58.7	17.4		X	X
2	58.5	57.8	56.3	25.8	X	X	
2	11.2	9.8	6.3	190.9	X		X
3	65.3	64.5	62.7	4.0	X	X	X

**Table 6:** Description of sensor parameters.

Senor Performance	Fx, Fy	Fz	Tx, Ty	Tz
Sensing Range	130 N	400 N	10 Nm	10 Nm
Resolution	1/20 N	1/10 N	1/400 Nm	1/400 Nm
Resonant Frequency	1250 Hz	940 Hz	940 Hz	1250 Hz

**Table 7:** Correlation analysis on drag coefficients in experimental data ($C_{D}$-Exp).

Sample 1	Sample 2	N	Correlation	*p*-Value
H	$C_{D}$-Exp	125	−0.435	0.000
TA	$C_{D}$-Exp	125	−0.847	0.000
LA	$C_{D}$-Exp	125	0.050	0.578
TA	H	125	0.074	0.410
LA	H	125	0.389	0.000
LA	TA	125	0.000	1.000

**Table 8:** Optimal subset of drag coefficient from experimental data ($C_{D}$-Exp).

Vars	R-Sq	R-Sq(adj)	R-Sq(pred)	Mallows’ CP	H	TA	LA
1	71.7	71.5	70.7	226.1		X	
1	18.9	18.3	16.4	873.2	X		
1	0.3	0.0	0.0	1102.0			X
2	85.6	85.4	84.9	57.5	X	X	
2	71.9	71.5	70.5	225.0		X	X
2	24.6	23.4	21.0	805.5	X		X
3	90.1	89.9	89.4	4.0	X	X	X

**Table 9:** Correlation analysis on lift coefficients in experimental data ($C_{L}$-Exp).

Sample 1	Sample 2	N	Correlation	*p*-Value
H	$C_{L}$-Exp	125	0.068	0.451
TA	$C_{L}$-Exp	125	−0.964	0.000
LA	$C_{L}$-Exp	125	0.153	0.089
TA	H	125	0.074	0.410
LA	H	125	0.389	0.000
LA	TA	125	0.000	1.000

**Table 10:** Optimal subset of lift coefficient from experimental data ($C_{L}$-Exp).

Vars	R-Sq	R-Sq(adj)	R-Sq(pred)	Mallows’ CP	H	TA	LA
1	93.0	93.0	92.7	96.0		X	
1	2.3	1.5	0.0	2910.7			X
1	0.5	0.0	0.0	2968.7	X		
2	95.3	95.3	95.1	25.7		X	X
2	95.0	94.9	94.7	37.1	X	X	
2	2.3	0.7	0.0	2912.5	X		X
3	96.1	96.0	95.8	4.0	X	X	X

## Data Availability

All data included in this study are available upon request by contacting the corresponding author.

## Supplementary Materials

The following supporting information can be downloaded at: https://www.mdpi.com/article/10.3390/biomimetics9020081/s1, Video S1: The DLTdv toolkit was used to process the crab video and obtain the posture data of the crab when walking; Video S2: Hydrodynamic simulation of the bionic crab shell model using STAR-CCM+ software; Video S3: An experiment with a crab shell model in a circulating water tank to obtain data on the lift and drag of the model at different water velocities; Video S4: The robot walking in the pool with the bionic crab shell at a given transverse angle. File S1: the 3D model file of the experimental platform, which contains components such as the circulating water tank, support frame, simulated bottom, attitude parameters adjustment device, the sensors, and the model.
